# A Comparative Analysis of COVID-19-Associated and Non-COVID-19-Associated Mucormycosis

**DOI:** 10.2174/0118715303335275250116055824

**Published:** 2025-02-19

**Authors:** Süheyla Kömür, Aslıhan Candevir, Ayşe Seza İnal, Ferit Kuşcu, Behice Kurtaran, Funda Memişoğlu, İlkay Karaoğlan, Yeşim Taşova

**Affiliations:** 1 Department of Infectious Diseases and Clinical Microbiology, Faculty of Medicine, Cukurova University, Adana, Turkey;; 2 Department of Infectious Diseases and Clinical Microbiology, İnönü University, Malatya, Turkey;; 3 Department of Infectious Diseases and Clinical Microbiology, Gaziantep University, Gaziantep, Turkey

**Keywords:** Corticosteroid, COVID-19, bone destruction, diabetes mellitus, mucormycosis, risk factor

## Abstract

**Background:**

COVID-19-associated Mucormycosis (CAM) has emerged as a significant complication during the COVID-19 pandemic. However, there is a lack of comprehensive comparative studies with non-COVID-associated mucormycosis (NCM).

**Objective:**

This study aims to compare the clinical characteristics, risk factors, and outcomes of CAM and NCM to enhance the understanding and management of these infections, particularly during the COVID-19 pandemic.

**Methods:**

A retrospective multicenter study was conducted at Cukurova University, Malatya İnönü University, and Gaziantep University. We analyzed and compared cases of CAM and NCM diagnosed between January 2018 and February 2022. Data were collected from the infectious diseases and clinical microbiology departments, including demographic details, underlying conditions, treatment regimens, and outcomes.

**Results:**

A total of 38 cases were analyzed, with 21 cases of COVID-19-associated mucormycosis (CAM) and 17 cases of non-COVID-19-associated mucormycosis (NCM). The key findings of the study were as follows: CAM was strongly associated with corticosteroid use (*p*<0.001) and diabetes (*p*=0.001), while NCM cases were more frequently linked to malignancy and neutropenia (*p*<0.05). Clinically, CAM cases had a higher incidence of cavernous sinus involvement and bone destruction (*p*=0.003) compared to NCM cases. However, there was no significant difference in overall survival between the CAM and NCM groups (*p*=0.201).

**Conclusion:**

The study highlights the critical role of corticosteroid use and diabetes as prominent risk factors for CAM. Timely diagnosis and intervention are essential to prevent severe complications, such as cavernous sinus involvement and bone destruction. These findings emphasize the need for tailored management strategies for CAM in the context of COVID-19, with particular attention to these risk factors.

## 1 INTRODUCTION

Since the initial emergence of SARS-CoV-2, approximately 800 million cases have been reported, resulting in the death of more than seven million infected individuals [1]. Although hyperinflammation due to excessive cytokine release is most commonly blamed for COVID-19-associated deaths, paradoxically, significant suppression of T, natural killer, and B lymphocytes is observed in the clinical presentation of the disease, which increases the patient’s susceptibility to secondary bacterial and fungal infections. Among the opportunistic infections, mucormycosis has received increasing attention [2]. There have been numerous reports of patients with COVID-19-associated mucormycosis (CAM). In the majority of these cases, the patients have also been diagnosed with diabetes mellitus and had a history of corticosteroid treatment for COVID-19. Rhino-orbital-cerebral mucormycosis is most frequently reported, with symptoms including facial pain, facial or orbital swelling, headache, and/or nasal eschar. Pulmonary mucormycosis infections have been identified in individuals with COVID-19 who exhibit unexplained worsening pulmonary status or complications. Gastrointestinal mucormycosis has also been reported. Reports suggest that the onset of mucormycosis symptoms usually occurs five to 14 days after admission for COVID-19, but there are reports of individuals receiving both diagnoses at the time of presentation [3]. The majority of reports are from India, but cases have been reported from all around the world [4]. The mucormycosis case-fatality rate in case series from India at 12 weeks was 45.7% [5]. The change in the incidence and risk factors related to mucormycosis infection, which has increased remarkably as the pandemic continues, has been a matter of curiosity.

There is a gap in knowledge regarding the differences between CAM and mucormycosis cases not associated with COVID-19 (NCM). Accordingly, this study, aimed to examine the characteristics of CAM and to compare the underlying conditions and outcomes between CAM and NCM.

## 2 MATERIALS AND METHODS

This retrospective, multicenter study included patients with CAM diagnosed between February 2020 and February 2022, and patients with non-COVID-19-associated mucormycosis NCM diagnosed between 2018 and 2020. The study was conducted across three centers: Cukurova University, Gaziantep University, and Inonu University.

### 2.1 Inclusion Criteria

Patients aged 18 years or older who met the diagnostic criteria for proven or probable mucormycosis, as outlined by the European Organization for Research and Treatment of Cancer/Mycoses Study Group (EORTC/MSG) guidelines, were included in the study.

- Proven mucormycosis was defined as a case with direct microscopic and/or culture-dependent microbiologic confirmation or histopathological evidence in a clinically or radiologically suspected patient.
- Probable mucormycosis was diagnosed based on the presence of diagnostic nasal endoscopy findings and/or radiological evidence that supported a clinical suspicion of mucormycosis.

CAM was diagnosed in patients who had a positive SARS-CoV-2 PCR (polymerase chain reaction) test at the time of or within 12 weeks before the diagnosis of mucormycosis, accompanied by related symptoms. NCM was diagnosed in patients who did not have a positive SARS-CoV-2 PCR test at the time of or within 12 weeks before the diagnosis of mucormycosis and lacked related symptoms.

### 2.2 Data Collection and Diagnosis

Data for both CAM and NCM patients were collected across all participating centers using a standardized case-record form. The diagnosis of COVID-19 was confirmed *via* PCR testing on nasopharyngeal and oropharyngeal swabs.

### 2.3 Study Objectives

The study aimed to compare CAM and NCM patients in terms of underlying conditions, clinical course of the disease, and outcomes.

### 2.4 Statistical Analysis

Data were compiled and managed using Microsoft Excel, and statistical analysis was performed using SPSS version 20.0. Descriptive statistics were reported as means with standard deviations for quantitative variables and as frequencies with percentages for categorical variables. To explore associations between variables, the Chi-square test was used for categorical data, while the unpaired t-test was employed for continuous variables. A *p*-value of 0.05 was considered statistically significant for all tests.

## 3 RESULTS

The study included 38 patients diagnosed with mucormycosis, of whom 21 (55%) had CAM and 17 (45%) had NCM. Of the cases, 22 (57%) were females, and the mean age was 57 years (Table **1**).

There were no statistically significant differences between the CAM and NCM groups in terms of age and gender (*p*>0.05 for both).

According to EORCT/MSG criteria, all of our cases had proven mucormycosis, and the diagnosis was confirmed by histopathology in patients with suspicious clinical and radiologic findings. A culture was not feasible for several reasons, including inappropriate sampling and the storage of samples until examined.

### 3.1 CAM Cases

 mean duration between COVID-19 and mucormycosis was 15.3 days (8–22 days). During the period of SARS-CoV-2 infection, 14 patients were monitored in outpatient clinics, three in clinics, and four in intensive care units. Nine patients did not require oxygen therapy, eight received oxygen *via* a reservoir mask, three received high-flow oxygen therapy, and one required mechanical ventilation. All patients received systemic corticosteroid therapy, either methylprednisolone or dexamethasone. Of these patients, 13 (61.9%) received high-dose pulse therapy. No other immunomodulatory agents, such as anakinra or tocilizumab, were administered.

Table **2** presents the predisposing factors for mucormycosis. The prevalence of diabetes was significantly higher in the CAM group than in the NCM group. Prior to hospitalization for COVID-19, five patients were administered insulin, and six were given oral antidiabetic drugs. Although five patients had a diabetes diagnosis, they were not receiving follow-up treatment. Furthermore, three patients were diagnosed with diabetes during their hospitalization for COVID-19. No NCM patients were newly diagnosed with diabetes.

The initial findings were similar in both groups, with the CAM group reporting periorbital edema and pain as the most common symptoms and the NCM group reporting facial edema and fever as the most common symptoms. With the exception of periorbital pain, which was significantly higher in the CAM group, the frequency of symptoms was not statistically different between the two groups (Table **3**).

All patients underwent paranasal tomography, as well as magnetic resonance imaging of the cranium and orbital cavity when cranial or orbital involvement was suspected. All patients presented with either rhino-orbital or rhino-orbito-cerebral forms of mucormycosis. The clinical forms were similar between the groups. However, there was a statistically significant increase in cavernous sinus involvement and bone destruction in the CAM group (Table **4**).

Endoscopic sinus surgery was performed on 84.2% (32) of the patients. In the CAM group, all patients received LAMB (*Liposomal* amphotericin *B*) except for one who refused parenteral treatment. Similarly, all patients in the NCM group received LAMB.

Of the 14 patients, six from the CAM group and eight from the NCM group died, resulting in a similar mortality rate in each group. The mean age of the deceased patients was 57.07 years (37–79), and their BMI (body mass index) was 27.3. There was no association between mortality and sex, age, diabetes, pulse corticosteroids, or neutropenia. The average time between the onset of mucormycosis and death was 31 days in the CAM group and 47 days in the NCM group. In all patients, the average time was 40.3 days, which did not differ significantly between the groups.

## 4 DISCUSSION

A significant concern during the COVID-19 pandemic was the increase in the amount of cases of mucormycosis worldwide, particularly in India [3, 6-8]. Mucormycosis is a significant health concern in Turkey as well, and there are numerous reports in the literature before and after the COVID-19 pandemic [9-11]. However, there are limited data on whether there are differences in the risk factors and clinical presentation of mucormycosis compared with the pre-pandemic period. In this study, the demographic data of patients were consistent with the published literature, with a similar mean age between CAM and NCM patients. However, unlike other studies, there was a predominance of females in this study, which may be attributed to the small number of patients overall.

The diagnosis of our patients was confirmed by histopathological and radiological examination in addition to clinical findings in both groups. For several reasons, including inappropriate sampling, storage of samples until examination, and laboratory regulations, culturing was not feasible. Due to the current frightening developments in the epidemiology of mucormycosis, diagnostic opportunities need to be improved. Moreover, species-level identification might be helpful for treatment management.

Muthu *et al.* defined “early CAM” as mucormycosis diagnosed simultaneously or within seven days of confirmation of COVID-19, with cases diagnosed up to three months later as “late CAM.” While the average time between COVID-19 and mucormycosis was 19.9 days in cases reported from India, a length of 17.2 days was reported in a study in other countries [3]. In this study, this period was found to be 15.8 days (8–22), similar to that in other countries. There was no CAM detected in the first seven days.

In this study, diabetes and corticosteroid use were found to be the most prominent risk factors for mucormycosis in patients with COVID-19. Based on the literature, the common underlying diseases in mucormycosis include diabetes mellitus with or without diabetic ketoacidosis, hematological malignancies, transplant recipients, corticosteroid therapy, and neutropenia [12, 13]. It has been observed that SARS-CoV-2 infection potentiates the facilitating effect of these factors [4, 14-19]. There has been speculation that in SARS-CoV-2 infection, the reduction in the absolute number of lymphocytes and T-cells predisposes to invasive mucormycosis [13]. Additionally, there may be changes in the distribution of the underlying risk factors due to the effect of SARS-CoV-2 infection and the medications used. Moreover, the distribution of mucormycosis risk factors varies based on geographical region. Diabetes is the main risk factor in India, with a higher rate than in other countries [4]. India also has the highest number of diabetics in the world, with a rate exceeding 30% of the population [19]. Sen *et al.* studied 2,826 patients with CAM from India, reporting that diabetes was present in 78% of the patients [18]. In our study, 90.5% (19) of CAM patients were observed to have diabetes, which was statistically higher than in the non-CAM group. Notably, three cases were diagnosed with diabetes during SARS-CoV-2 infection. New-onset diabetes has been reported in 20.6% of patients with mild-to-moderate COVID-19 [20]. Indeed, a possible association between SARS-CoV-2 infection and the development of diabetes has been suggested in the literature. ACE 2 overexpression in the pancreatic ductal epithelium and at the microvascular level leads to infection of pancreatic endocrine cells with SARS-CoV-2 and diabetogenic clinical findings through cytokine-mediated cell damage [14-17].

In the NCM group, cancer and neutropenia were identified as the main risk factors, which were significantly higher in the NCM group than in the CAM group. Higher rates of hematological cancer and organ transplantation were reported in CAM cases in countries other than India [7]. Changes in the management of cancer treatment during the pandemic period may have caused this situation. A global survey indicated a reduction in cancer treatments (69%), the use of fewer immunosuppressive agents (50%), and treatment breaks (14%) during the COVID-19 pandemic [21].

Corticosteroids are potent immunosuppressants and are a major predisposing factor for mucormycosis. Hyperglycemia induced by corticosteroids further increases the susceptibility to CAM [22]. Corticosteroid therapy has been used to treat severe SARS-CoV-2 infections because it reduces the duration of invasive mechanical ventilation and hospital mortality [23]. A meta-analysis revealed that administration of systemic corticosteroids resulted in lower 28-day all-cause mortality compared with usual care [24]. The use of glucocorticoids causes increased insulin resistance, suppression of macrophage functions, difficulty in glycemic control, immunosuppression with sequestration of CD4 T- lymphocytes from the reticuloendothelial system, and inhibition of cytokine transcription [22]. In a prospective study of patients with hematologic malignancies, De León-Borrás *et al.* showed that the use of corticosteroids increased the risk of developing invasive fungal infections by 3.33 times [25]. In non-COVID-19 patients, prolonged, high-dose systemic use of corticosteroids for more than three weeks is a known risk factor for mucormycosis. The updated COVID-19 guidelines recommend the use of intravenous methylprednisolone (0.5–1 mg/kg) or an equivalent dose of dexamethasone for moderate and severe disease for a duration of 5–10 days [26]. All of our patients with CAM had a history of corticosteroid use due to the diagnosis of COVID-19. Thirteen of 21 (61.9%) patients had taken pulse steroids. According to the literature, 80.3% of CAM patients have used corticosteroids in India, compared to 71.1% in the rest of the World [4, 27]. A systematic review and meta-analysis published in February 2021 stated that immunomodulatory therapies, particularly tocilizumab, show promise in treating severe COVID-19 [28]. There have been some reports of CAM in patients treated with anakinra or tocilizumab [29]. None of our patients had taken anakinra or tocilizumab, which can be attributed to the difficulty of access at that time. In addition, difficulty in accessing supportive treatments may be the reason for the shift to corticosteroid use. There is a wealth of data on the inappropriate use of corticosteroids. In our study, although three patients were followed at home, oral corticosteroid treatment was given. Unlike the CAM group, patients in the NCM group did not use steroids.

In a study with 2,826 patients with CAM, the most frequently reported symptoms were orbital/facial pain (23%), edema (21%), vision loss (19%), ptosis (11%), and nasal congestion (9%) [18]. In our study, the most common initial findings in the CAM group were periorbital edema and pain, while they were facial edema and fever in the NCM group. There was no statistically significant difference between the two groups in terms of the frequency of findings. However, periorbital pain was significantly more common in the CAM group.

During the COVID-19 pandemic, rhino-orbital or rhino-orbital-cerebral mucormycosis were the most frequently reported disease forms worldwide [30]. This may be due to the difficulties in diagnosing other conditions, such as pulmonary mucormycosis, which is particularly challenging and might have often been missed during this period [6]. In addition, pulmonary and disseminated disease is reported more commonly outside of India. In this study, the clinical forms were rhino-orbital or rhino-orbital-cerebral mucormycosis in all of the patients, similar to India. This may be due to the similar demographic characteristics and risk factors of the Indian population and our sample.

Differences in the characteristics of involvement were observed between the CAM and NCM patient groups. Patients with CAM had significantly higher rates of bone destruction and cavernous sinus involvement compared to NCM patients. Although the reason for this finding is not known exactly, it could have been the result of the addition of viral infection to other contributing factors.

The surgical intervention rate was similar in both groups. Orbital exanthema was applied to six CAM patients and three NCM patients. The crucial role of surgery in the management of mucormycosis has been emphasized in several studies, which demonstrated the impact of early and complete surgical debridement on patient survival [31].

The mortality rates of mucormycosis varied from 40 to 96%, depending on the affected site and underlying conditions [32, 33]. Before the COVID-19 pandemic, mucormycosis mortality was 52.9% in our series [11]. Increased mortality rates were reported during the pandemic, especially in India. In a review, all-cause mortality occurred in 39 (49%) of 80 patients from 18 countries [3].

In this study, the mortality ratio was similar between the NCM and CAM groups. In CAM patients, the mortality rate was 33.3%, which is lower than in other countries. This can be attributed to the fact that all of our cases had the rhino-orbital-cerebral disease form and had access to LAMB and the possibility of surgery. Many studies have suggested that, even with COVID-19, early intravenous antifungal treatment and surgical debridement were associated with favorable outcomes [31, 33, 34].

Although mucormycosis is rare, it has high morbidity and mortality rates similar to those before the COVID-19 pandemic [33]. The risk factors for CAM may differ and may be more aggressive. During SARS-CoV-2 infection, especially in the presence of diabetes and corticosteroid therapy, mucormycosis should be considered. Early diagnosis and treatment, including surgical intervention, are important for favorable outcomes [34].

This study has some limitations because of its retrospective design and the small sample size, which reduces the generalisability and statistical power of the findings. Further research should, therefore, be conducted using larger, multicentre, prospective studies to confirm these findings.

## CONCLUSION

This study highlights the distinct risk factors and clinical features of CAM compared to NCM. CAM was strongly linked to corticosteroid use and diabetes, with higher rates of severe complications like cavernous sinus involvement and bone destruction. While overall survival did not differ significantly between the two groups, early diagnosis and targeted intervention are crucial for managing CAM, particularly in the context of COVID-19. These findings emphasize the need for tailored treatment strategies focusing on controlling underlying risk factors.

## Figures and Tables

**Table 1:** Baseline features of patients with CAM and NCM.

**Variables**	**CAM, n=21**	**NCM, n=17**	**Total, n=38**	***p***
**Mean age**	56.3	58	57.08	0.81
**M/F**	9/12	7/10	16/22	0.59

**Table 2:** Underlying conditions of patients.

**Underlying Condition**	**CAM** **n (%)**	**NCM** **n (%)**	**Total** **n (%)**	***p***
**Diabetes**	19 (90.5)	6 (35.3)	25	<0.001
**Ketoacidosis**	3 (14.3)	0	3	0.158
**Hypertension**	8 (38.1)	2(11.8)	10	0.070
**Chronic renal failure**	5 (23.8)	1(5.9)	6	0.145
**Malignancy**	3 (14.3)	12 (70.6)	15 (39.5)	0.001
**Chronic renal failure**	5 (23.8)	1(5.9)	6	0.145
**Corticosteroids**	21(100)	0	21	0.001
**Pulse corticosteroids**	13 (61.9)	-	6	-
**Neutropenia**	1(4.8)	10(58.8)	11(28.9)	0.001
**Mean body mass index (kg/m^2^)**	30.1	25.7	28.13	0.14

**Table 3:** The initial findings of patients.

**Findings**	**CAM** **n(%)**	**NCM** **n(%)**	**Total** **n(%)**	***p***
**Fever**	7 (33.3)	11 (64.7)	18(47.4)	0.05
**Facial oedema**	13(61.9)	14(82.4)	27(71.1)	0.15
**Periorbital edema**	15 (71.4)	7 (41.2)	22 (57.9)	0.06
**Periorbital pain**	15 (71.4)	5 (29.4)	20 (52.6)	0.01
**Palatal ulcer**	11(52.4)	8(47.1)	19(50)	0.5
**Toothache**	8(38.1)	5(29.4)	13(34.2)	0.41
**Unconsciousness**	3(14.3)	2(11.8)	5(13.2)	0.60
**Headache**	15(71.4)	8(47.1)	23(60.5)	0.11

**Table 4:** Clinical presentation, surgical interventions, and outcome.

** Clinical Finding **	** CAM ** (n/%)	** NCM ** (n/%)	** Total ** (n/%)	***p***
Rhino-orbital	9 (42.9)	12(70.6)	21(55.3)	0.083
Rhino-orbito-serebral	12(57.1)	5 (29.4)	17 (44.7)	
Bone destruction	20 (95.2)	9(52.9)	29 (76.3)	0.003
Cavernous sinus involvement	14 (66.7)	3 (17.6)	17 (44.7)	0.003
Surgical intervention	17 (81)	15 (88.2)	32 (84.2)	0.440
Orbital exenteration	6 (28.5)	3 (17.6)	9 (23.6)	0.38
Exitus	7 (33.3)	8 (47.1)	15 (39.5)	0.201

## Data Availability

The data and supportive information are available within the article.

## LIST OF ABBREVIATIONS

CAM: COVID-19-Associated Mucormycosis
NCM: Non-COVID-Associated Mucormycosis
EORTC/MSG: European Organization for Research and Treatment of Cancer/Mycoses Study Group

## STANDARDS OF REPORTING

STROBE guidelines were followed.
